# Natural alleles of the clock gene *timeless* differentially affect life-history traits in *Drosophila*

**DOI:** 10.3389/fphys.2022.1092951

**Published:** 2023-01-10

**Authors:** Gabriele Andreatta, Sara Montagnese, Rodolfo Costa

**Affiliations:** ^1^ Department of Biology, University of Padua, Padua, Italy; ^2^ Max Perutz Laboratories, University of Vienna, Vienna, Austria; ^3^ Department of Medicine, University of Padua, Padua, Italy; ^4^ Chronobiology, Faculty of Health and Medical Sciences, University of Surrey, Guildford, United Kingdom; ^5^ Institute of Neuroscience, National Research Council (CNR), Padua, Italy

**Keywords:** circadian clock, timeless, developmental time, early-life fecundity, seasonality, photoperiodism, reproductive dormancy

## Abstract

Circadian clocks orchestrate a variety of physiological and behavioural functions within the 24-h day. These timekeeping systems have also been implicated in developmental and reproductive processes that span more (or less) than 24 h. Whether natural alleles of cardinal clock genes affect entire sets of life-history traits (i.e., reproductive arrest, developmental time, fecundity), thus providing a wider substrate for seasonal adaptation, remains unclear. Here we show that natural alleles of the *timeless* (*tim*) gene of *Drosophila melanogaster*, previously shown to modulate flies’ propensity to enter reproductive dormancy, differentially affect correlated traits such as early-life fecundity and developmental time. Homozygous flies expressing the shorter TIM isoform (encoded by the *s-tim* allele) not only show a lower dormancy incidence compared to those homozygous for *ls-tim* (which produce both the short and an N-terminal additional 23-residues longer TIM isoform), but also higher fecundity in the first 12 days of adult life. Moreover, *s-tim* homozygous flies develop faster than *ls-tim* homozygous flies at both warm (25°C) and cold (15°C) temperatures, with the gap being larger at 15°C. In summary, this phenotypic analysis shows that natural variants of *tim* affect a set of life-history traits associated with reproductive dormancy in *Drosophila*. We speculate that this provides further adaptive advantage in temperate regions (with seasonal changes) and propose that the underlying mechanisms might not be exclusively dependent on photoperiod, as previously suggested.

## Introduction

Maximising survival and reproductive success require synchronization of physiological and metabolic processes with environmental conditions that change in a rhythmic fashion, the day/night cycle representing the most prominent example. As the ability to anticipate such predictable environmental changes is likely to be adaptive, organisms have evolved time-keeping systems which allow better physiological, metabolic and behavioural preparation for the upcoming changes, and the opportunities and challenges that accompany them. These endogenous entrainable circadian clocks consist of molecular oscillators located in many cells and coordinated in animals by master clocks residing in the brain (Peschel and Helfrich-Förster, 2011; Takahashi, 2017). In commonly utilised animal models, from insects to mammals, the genetic architecture of the circadian clocks is well understood, as are the molecular dynamics coordinating a variety of fundamental biological processes with a ∼24 h periodicity (Peschel and Helfrich-Förster, 2011; Takahashi, 2017). However, a growing body of evidence shows how elements of the circadian clock machinery also play a role in orchestrating rhythmic processes with periods shorter or longer than 24 h, such as circatidal and circalunar/circannual ones, respectively (Reppert et al., 2015; Andreatta and Tessmar-Raible, 2020; Saunders, 2020). Moreover, circadian clocks – or some of their specific components – have been linked to the timing of egg-to-adult development in different experimental setups and organisms, including *D. melanogaster* and *Caenorhabditis elegans* (Kyriacou et al., 1990; Banerjee et al., 2005; Edelman et al., 2016; Olmedo et al., 2017; Srivastava et al., 2018). For instance, *Drosophila period* mutants with 19 h and 28 h circadian free-running periods (called *per* ^*S*^ and *per* ^*L*^) show shorter and longer pre-adult development, respectively (Kyriacou et al., 1990; Srivastava et al., 2018). In turn, artificial selection for short and long pre-adult developmental time in the melon fly *Bactrocera cucurbitae* resulted in circadian periods of ∼22.5 h and up to ∼31 h, respectively, indicating the existence of a relationship between developmental time and circadian periodicity (Shimizu et al., 1997). Also supporting a connection between the circadian clock and developmental processes, the duration of pre-adult development under LL and short light/dark (LD) cycles (LD 10:10) is shorter compared to longer LD cycles (LD 12:12, LD 14:14) and DD (Yadav et al., 2014). In line with this, selection for early or late eclosion, which is also modulated by the circadian clock (Myers et al., 2003; Mark et al., 2021), results in flies with faster and slower pre-adult development, respectively (Kumar et al., 2006). Finally, circadian clock genes and neurons are pivotal for the regulation of reproductive features such as fecundity (Beaver et al., 2003) and dormancy in *Drosophila* (Saunders et al., 1989; Sandrelli et al., 2007; Tauber et al., 2007; Nagy et al., 2019).

Despite advances in the understanding of the circadian implications of developmental and reproductive phenotypes, whether natural variants of core clock genes affect specific life-history traits or modulate developmental/reproductive trajectories remains unclear. Reproductive dormancy represents an interesting case study as: 1) it involves complex endocrine and organ crosstalk (Richard et al., 2001; Kubrak et al., 2014; Schiesari et al., 2016; Andreatta et al., 2018; Nagy et al., 2019), and 2) its incidence has been shown to co-vary with other life-history traits in natural populations (Schmidt et al., 2005a; 2005b; Schmidt and Paaby, 2008). In *Drosophila*, this overwintering strategy implies the arrest (or slowing) of gonads maturation (Saunders et al., 1989; Kubrak et al., 2016; Schiesari et al., 2016; Zonato et al., 2017), which ultimately postpones reproduction. Interestingly, two natural alleles at the *timeless* (*tim*) locus, one of the core circadian clock genes (Sehgal et al., 1994), have been shown to differentially affect dormancy incidence in populations across Europe (Tauber et al., 2007). Homozygous individuals for the *ls-tim* variant exhibit higher propensity to enter reproductive dormancy compared to their counterparts carrying the *s-tim* allele (Tauber et al., 2007). The *ls-tim* allele, which has originated more recently (300–3000 years ago) and spread by directional selection (Zonato et al., 2018), generates both a long (L-TIM_1421_) and a shorter protein (S-TIM_1398_), whereas the only TIM product of *s-tim* flies is the shorter isoform. The two isoforms are created by an insertion of a *G* nucleotide in position 294 of the *ls-tim* sequence that leads to the synthesis of L-TIM from an upstream *AUG.* Absence of this *G,* generates a stop codon 19 codons after the first *AUG,* but a second downstream *AUG* generates S-TIM (Rosato et al., 1997; Tauber et al., 2007). Thus, L-TIM and S-TIM differ by the presence of an additional N-terminal 23 amino acids. Mechanistically, this additional portion of the TIM protein seems to reduce the affinity for CRYPTOCHROME (CRY, which mediates the light-dependent degradation of TIM), thus resulting in dampened photosensitivity of the circadian clock in *ls-tim* flies (Sandrelli et al., 2007; Deppisch et al., 2022). Recent evidence shows how *ls-tim* flies (but not *s-tim* flies) can still synchronize to temperature cycles in constant light, a condition reminiscent of the extremely long photoperiods characterizing Summer at Northern latitudes (Lamaze et al., 2022). In this paper we provide robust phenotypic evidence indicating that the presence of *s-tim* or *ls-tim* alleles not only affects the propensity to enter reproductive dormancy (Tauber et al., 2007) but also several life-history traits such as early-life fecundity and developmental time. This suggests that the polymorphism at the *tim* locus could influence the entire *Drosophila* developmental trajectory, providing a powerful hub for seasonal adaptation.

## Materials and methods

### Fly stocks and maintenance

Fly stocks were maintained at 23°C in a 12:12 h light/dark (LD) cycle prior to the experiments. For both stock maintenance and the experimental setups described below, a standard yeast-sucrose-cornmeal diet was used (Andreatta et al., 2018). An isofemale line derived from a population collected in Houten, the Netherlands, was selected as our study model (Tauber et al., 2007). Using PCR genotyping combined with classical genetic crossing methods, the *ls-tim* and *s-tim* alleles segregating within this isofemale line were made homozygous in separate fly stocks (Gesto, 2010). By using this strategy, the resulting genetic background of the two homozygous *ls-* and *s-tim* flies is expected to be highly homogeneous.

### Reproductive dormancy assays

To test the incidence of reproductive dormancy in the two lines, we used a previously published protocol (Schiesari et al., 2016; Andreatta et al., 2018; Nagy et al., 2019). Briefly, larvae were reared under standard conditions at 23°C and LD 12:12 until eclosion. Newly eclosed virgin flies were collected (∼60 females and 60 males *per* replicate) within 5 h of eclosion, and rapidly exposed to low temperature (12°C) and short (LD 8:16) or long (LD 16:8) photoperiods for 11 days. Reproductive dormancy was defined as the complete absence of vitellogenesis (i.e. all oocytes at stages ≤7), examining all ovarioles in both ovaries of each specimen (Saunders et al., 1989; Tauber et al., 2007; Schiesari et al., 2016). Five biological replicates (∼60 females each, ∼300 flies in total) were analysed for every homozygous genotype (*s-tim* and *ls-tim*) and experimental condition (LD 8:16 and LD 16:8). Dormancy levels are presented as percentage of dormant females. Percentage data were arcsine square-root transformed to be analysed by one-way ANOVA (*post hoc*: Tukey test) (Tauber et al., 2007; Schiesari et al., 2016; Andreatta et al., 2018) using GraphPad Prism 9.0.0.

### Early-life fecundity assessment

Female fecundity was measured as the number of eggs laid. Eleven virgin females for each genotype were collected and individually placed in vials, where they were allowed to mate with two males each for the following 2 days. Then, females were isolated from males and the number of eggs laid recorded for the following 12 days, changing the food medium (standard yeast-sucrose-cornmeal) every day. The entire experiment was conducted at 23°C and under LD 12:12. Data are presented as number of eggs laid/day, as well as total number. Statistical significance was assessed using the Kolmogorov-Smirnov and t test, respectively.

### Developmental time analysis

To assess the developmental time of *Hu s-tim* and *ls-tim* wild-type homozygous flies we used the protocol described in (McBrayer et al., 2007). Fertilized eggs were collected on peach juice agar plates. Prior to the collection of suitable eggs, females were allowed to lay potentially held eggs for 1 h. Then, females were allowed to lay eggs in 2 h intervals for 6 h in total (3 biological replicates). From each of these three egg deposition series, 20 first-instar larvae were collected and placed into each of 10 vials with 2.5 ml of standard yeast-sucrose-cornmeal food (∼200 individuals for each of the three biological replicates, and for both genotypes). Larvae were raised in a dedicated incubator set at 25°C and LD 12:12. Larvae development was monitored till pupariation with 2 h intervals. For the second set of experiments, we used the same procedure described above, except that the incubator temperature was set at 15°C, and pupariation scored every 6 h. We opted for 15°C as pupal mortality increases dramatically at temperatures lower than 15°C (David and Clavel, 1967). Data are shown as 1) % of pupariated larvae over time; 2) number of pupariating individuals at each timepoint; 3) average hour after egg deposition (hours AED); 4) % increase in egg-to-adult developmental time. To calculate the % increase in *ls-tim* developmental time, the timespan of biological replicate one from *s-tim* homozygous flies, at 25°C and 15°C, respectively, was used as reference. Comparisons between developmental times (hours after egg deposition, hours AED) and relative % were performed by the Mann-Whitney U and t test, respectively.

### Fly weight

Fly weight was assessed in flies emerging from the developmental time experiment performed at 15°C, in which the quantified developmental delay of *ls-tim* flies was larger (36.36 h) than that at 25°C (13.52 h). A ∼30 h developmental delay has been previously shown to associate with a detectable increase in fly weight (Layalle et al., 2008). Thus, based on this piece of literature, we expected the effect on weight, if any, to be more obvious at 15°C than at 25°C. Twenty first-instar larvae were reared in every vial with standard yeast-sucrose-cornmeal food. Flies were collected over a 24 h timespan and weighted in batches of 10 (3 biological replicates and 30 individuals per genotype and sex, with 120 flies in total), and the average weight per fly determined (McBrayer et al., 2007). Data are reported as average weight (mg), and % increase in adult weight. In the latter case, to calculate the % increase in weight in *ls-tim* homozygous flies, the weight of biological replicate one from *s-tim* homozygous males and females, respectively, was used as reference.

## Results

### *Drosophila* females homozygous for the *ls-tim* allele exhibit higher dormancy propensity and reduced early-life fecundity

**FIGURE 1:**
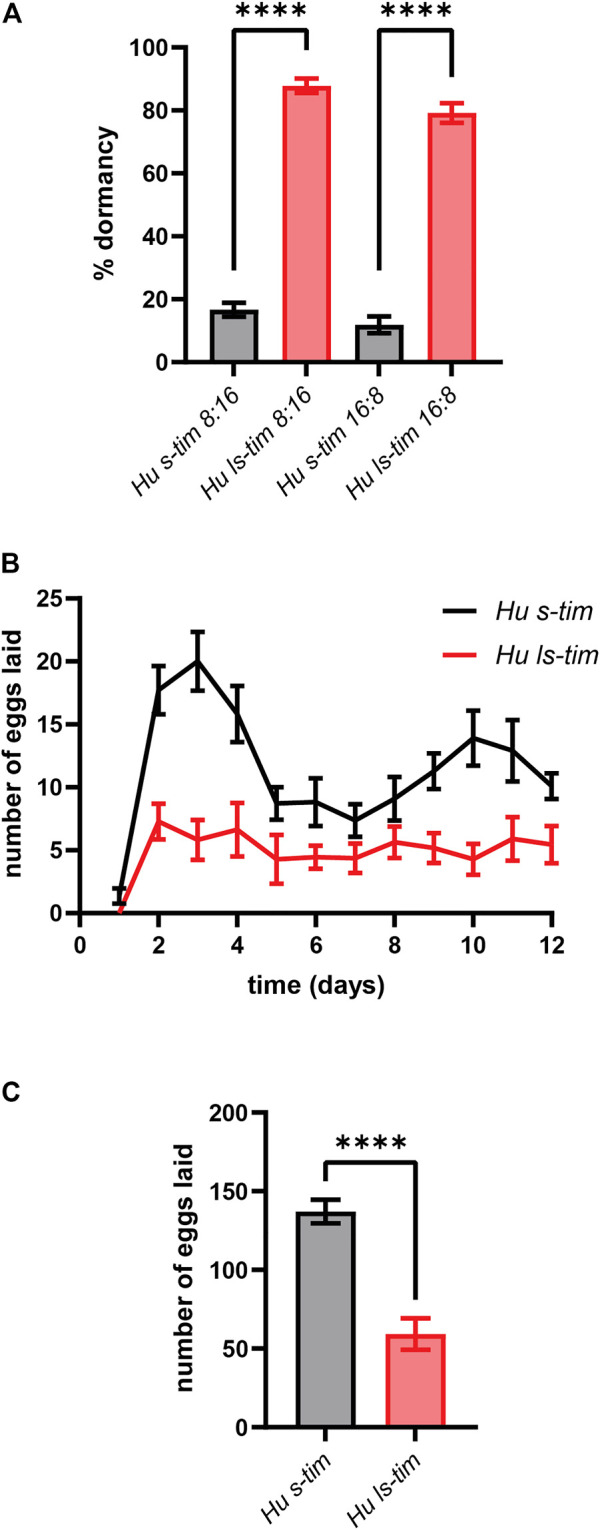
*ls-tim* homozygous females show reduced early-life fecundity compared to their *s-tim* homozygous counterparts. The lines used in this study are called Houten (Hu) *s-tim* and *ls-tim*, from the location in the Netherlands where the natural population of origin was collected. **(A)** Proportions (±SEM) of *s-tim* (black) and *ls-tim* (red) homozygous females in reproductive dormancy after 11 days at 12°C under both short (LD 8:16) and long (LD 16:8) photoperiods. Statistical analysis was performed using one-way ANOVA (*post hoc*: Tukey test), df = 19, ****p < 0.0001. **(B)** Average number of eggs laid daily (±SEM) by *s-tim* and *ls-tim* homozygous females at 23°C (LD 12:12) during the first 12 days of adult life. Statistical analysis was performed using the Kolmogorov-Smirnov test, ****p < 0.0001. **(C)** Total number of eggs laid (±SEM) by *s-tim* and *ls-tim* homozygous females during the 12 days considered. Statistical analysis was performed by t test, df = 20, ****p < 0.0001.

Under both short (LD 8:16) and long (LD 16:8) photoperiods - mimicking winter- and summer-like light conditions, respectively - the *ls-tim* homozygous flies showed a significantly higher proportion of dormant females compared to the *s-tim* homozygous flies (one-way ANOVA, *post hoc*: Tukey test, df = 19, *p* < 0.0001) (Figure 1A), as expected (Tauber et al., 2007). Interestingly, both *s-tim* and *ls-tim* flies exposed to short photoperiods exhibited a slightly higher propensity to enter dormancy (+4.8% and +8.6%, respectively) compared to their counterparts exposed to long photoperiods, although no significant difference was observed (Figure 1A). In *Drosophila*, reproductive dormancy implies an arrest/slowing of ovarian development (Saunders et al., 1989; Schiesari et al., 2016; Zonato et al., 2017). We thus wondered whether *s-tim* and *ls-tim* homozygous females showed signs of distinct reproductive profiles even in conditions that do not elicit dormancy. Egg laying was therefore monitored for the first 12 days after mating of newly eclosed flies reared and maintained at 23°C and LD 12:12, and the number of eggs laid was determined daily. Overall, *s-tim* females were found to lay a higher number of eggs compared to *ls-tim* females (Kolmogorov-Smirnov test, *p* < 0.0001) (Figure 1B). While egg laying started synchronously in *s-tim* and *ls-tim* females, it then showed larger peaks around days 2–4 and 9–12 in *s-tim* flies (Figure 1B). Finally, the overall number of eggs laid by *ls-tim* homozygous females was significantly reduced compared to their *s-tim* counterparts over the time span considered (t test, df = 20, *p* < 0.0001) (Figure 1C).

### *Timeless* alleles differentially affect developmental time at different temperatures

**FIGURE 2:**
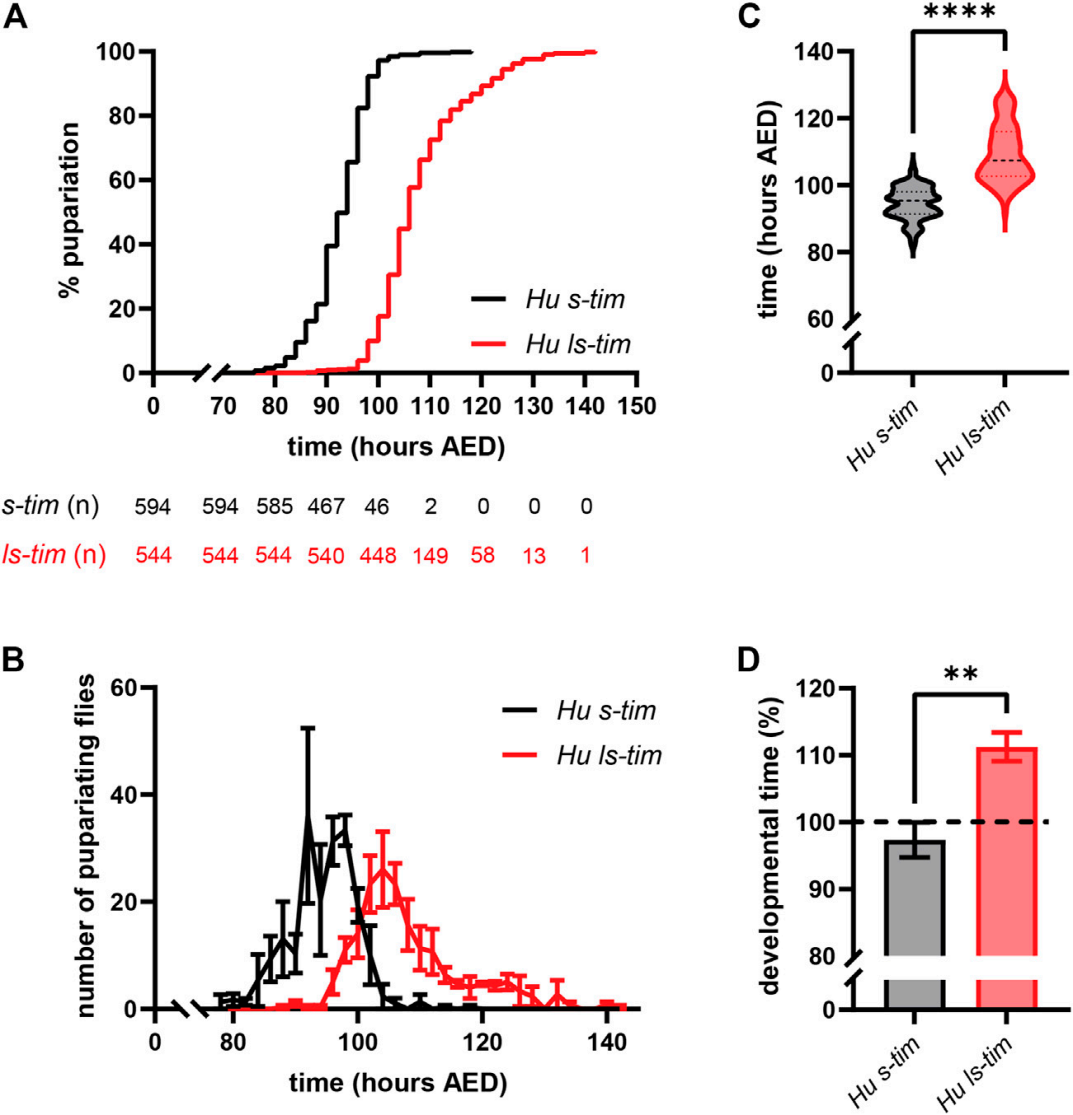
*ls-tim* homozygous flies’ egg-to-adult development is delayed compared to that of *s-tim* homozygous flies at 25°C. **(A)** Pupariation curves of *s-tim* (black) and *ls-tim* (red) homozygous larvae, pupariating over time at 25°C (LD 12:12). The interval between the time of egg deposition and pupariation was used to quantify developmental time. Log-rank (Mantel-Cox) test, *p* < 0.0001. AED: After Eggs Deposition. The number of residual, non-pupariated larvae from each genotype at different timepoints is reported below the graph. **(B)** Average number (±SEM) of pupariating *s-tim* and *ls-tim* homozygous larvae at 25°C over-time. **(C)** Egg-to-adult developmental time of *s-tim* and *ls-tim* homozygous flies at 25°C. Statistical analysis of developmental time (hours after egg deposition, AED) was performed by Mann-Whitney U test, ****p < 0.0001. **(D)** % increase in the developmental time of *ls-tim* flies at 25°C compared to their *s-tim* counterpart used as reference (t test, df = 4, **p < 0.01).

The time from egg to pupariation was monitored in individuals reared in density-controlled conditions at 25°C and LD 12:12. s*-tim* homozygotes developed significantly faster compared to *ls-tim* homozygotes [Log-rank (Mantel-Cox) test, *p* < 0.0001] (Figure 2A). The pupariation profiles showed similar dynamics, but were shifted later in *ls-tim* flies, with a longer tail of the pupariation curve (Figure 2B). The number of individuals reaching pupariation peaked at 104 h from egg deposition in *ls-tim* flies compared to 92–98 h in *s-tim* flies (Figure 2B). The average difference in developmental time between the two homozygous genotypes was quantified as 13.52 h (Mann-Whitney U test, *p* < 0.0001) (Figure 2C), with a significant % increase in *ls-tim* flies developmental time of ∼14% (t test, df = 4, *p* < 0.01) (Figure 2D).

**FIGURE 3:**
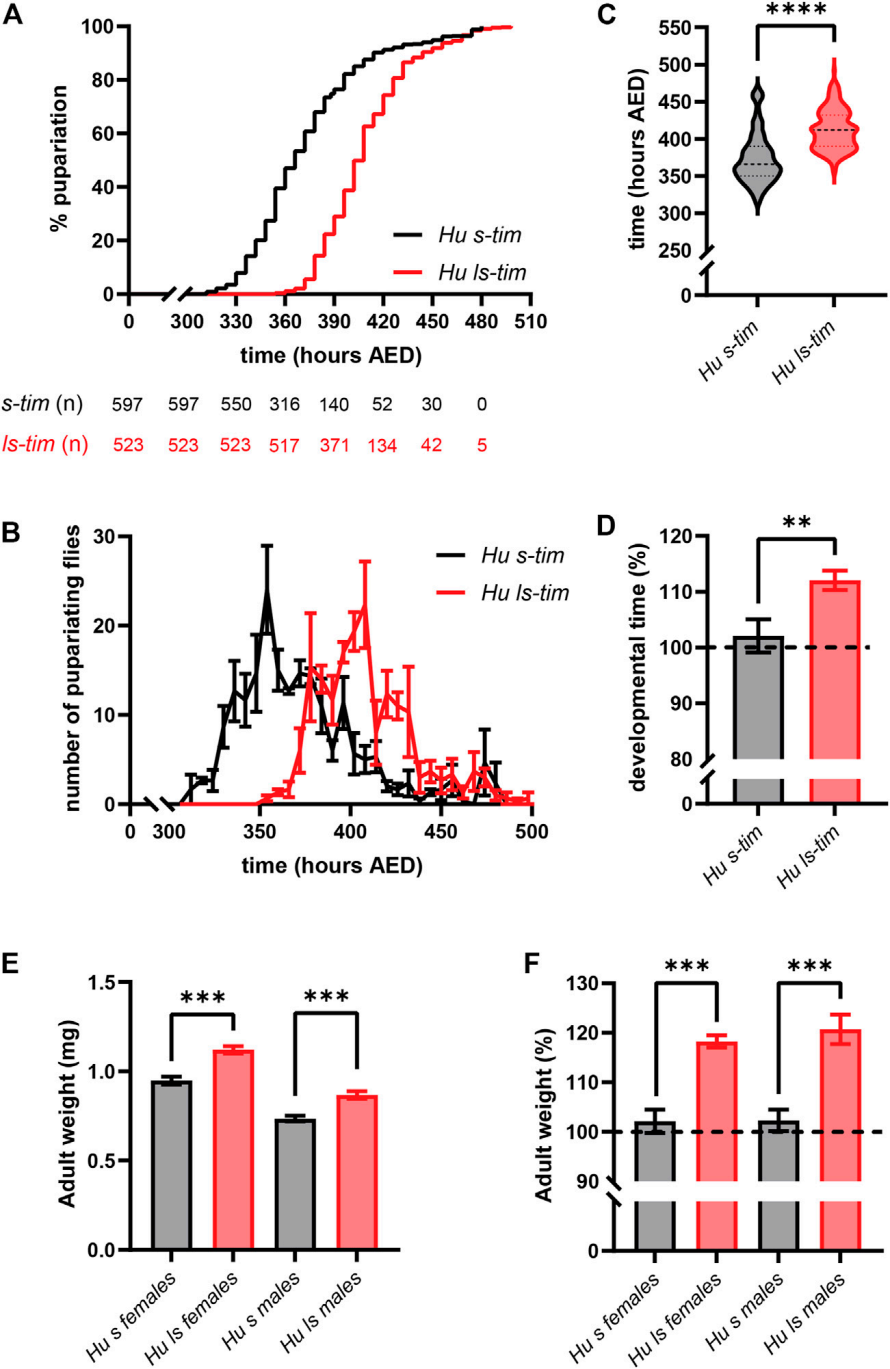
Differences in developmental time between *s-tim* and *ls-tim* homozygous flies increase at lower temperature and lead to changes in the size of emerging flies. **(A)** Pupariation curves of *s-tim* (black) and *ls-tim* (red) homozygous larvae pupariating over time at 15°C (LD 12:12). The time spanning between egg deposition and pupariation was defined as developmental time. Log-rank (Mantel-Cox) test, *p* < 0.0001. AED: After Eggs Deposition. The number of residual, non-pupariated larvae from each genotype at different timepoints is reported below the graph. **(B)** Average number (±SEM) of pupariating *s-tim* and *ls-tim* homozygous larvae at 15°C over time. **(C)** Egg-to-adult developmental time of *s-tim* and *ls-tim* homozygous flies at 15°C. Statistical analysis of developmental time (hours after egg deposition, AED) was performed by Mann-Whitney U test, ****p < 0.0001. **(D)** % increase in the developmental time of *ls-tim* flies at 15°C compared to *s-tim* individuals used as reference (t test, df = 4, **p < 0.01. **(E)** Adult weight of *s-tim* and *ls-tim* homozygous males and females emerging in a 24 h window. For every biological replicate, flies were weighted in batches of 10, and the average weight (±SD) of each fly determined and plotted. Statistical analysis was performed by t test, df = 4, ***p < 0.001. **(F)** Increase in adult weight in *ls-tim* homozygous males and females expressed as % (±SD) of the weight of their *s-tim* counterparts. Statistical analysis was performed by t test, ***, df = 4, *p* < 0.001.

The hypothesis that different developmental times in *s-tim* and *ls-tim* homozygous flies are modulated by lower temperatures (15°C), which resemble more closely those triggering dormancy (12°C–14°C), was tested. We opted for 15°C as below this temperature pupal mortality increases dramatically (David and Clavel, 1967). Even at low temperatures, *ls-tim* flies developed significantly slower from egg to pupariation compared to their *s-tim* counterparts [Log-rank (Mantel-Cox) test, *p* < 0.0001] (Figure 3A). However, the average gap between the two homozygous genotypes, quantified as 13.52 h at 25°C, increased to 36.36 h at 15°C (Mann-Whitney U test, *p* < 0.0001) (Figure 3C). In spite of the observed temperature-dependent effect, the average % increase in the developmental time of *ls-tim* flies was 10% (t test, df = 4, *p* < 0.01), thus proportional to the increase at 25°C (Figure 3D). As observed at warmer temperatures, the pupariation profiles of *s-tim* and *ls-tim* lines were similar at 15°C, although in this case the former showed a slightly longer tail, with peaks occurring at 354 h and 402 h after egg deposition (AED), respectively (Figure 3B). These data suggest that the difference in developmental time of flies bearing different *tim* alleles is more prominent at lower temperatures. A prolonged larval development is known to cause an increase in fly size and weight (Layalle et al., 2008), which may have an adaptive value at higher latitudes for species experiencing colder temperatures, including diapausing/dormant ones (Chown and Gaston, 2010; Stillwell, 2010; Hahn and Denlinger, 2011). Also, in line with this hypothesis, both *ls-tim* homozygous males and females developing at 15°C showed a significant increase in weight compared to their *s-tim* homozygous counterparts (t test, df = 4, *p* < 0.001) (Figure 3E), with a ∼20% gain in both sexes (t test, df = 4, *p* < 0.001) (Figure 3F).

## Discussion

By comparing the early-life fecundity and developmental time correlates of *s-tim* and *ls-tim* homozygous flies, we have documented a profound impact of natural *tim* alleles on *Drosophila* life-history. *ls-tim* homozygous females not only showed higher propensity to enter reproductive dormancy (Tauber et al., 2007) but also reduced early-life fecundity, which suggests a dampened pace of gonadal maturation even in environmental conditions that do not elicit dormancy. Moreover, *ls-tim* homozygous flies show a slower developmental progression from eggs to pupariation compared to *s-tim* flies, with the gap increasing when the lower temperature is closer to that inducing dormancy. While the strategy utilised to obtain the two homozygous *ls-* and *s-tim* line is expected to result in a highly homogeneous genetic background, flies from the two lines could still carry some genetic variability. Thus, it cannot be excluded that residual polymorphisms, in particular in linkage disequilibrium with the alleles at the *tim* locus, could have affected the investigated phenotypes.

Under the experimental conditions used in previous studies of ours (Schiesari et al., 2016; Andreatta et al., 2018), the effects of the *s-tim - ls-tim* polymorphism were stronger than those potentially linked to the *couch potato* (*cpo*) gene, where distinct alleles have been associated with dormancy incidence in *D. melanogaster* populations from North America (Schmidt et al., 2008). Moreover, *cpo* variants show negligible effects on dormancy inducibility in European populations, in which the *In(3R)Payne* inversion (where *cpo* lies) is rare and not clinally distributed (Zonato et al., 2016). For these reasons, we focused exclusively on the *s-tim* - *ls-tim* polymorphism. Several lines of evidence suggest that TIM is key to the interface between environmental cues (such as photoperiod and temperature), clock functioning and seasonal adaptation (Majercak et al., 1999; Wijnen et al., 2006; Boothroyd et al., 2007; Montelli et al., 2015; Anduaga et al., 2019; Foley et al., 2019; Abrieux et al., 2020; Lamaze et al., 2022). For instance, the different propensity to undergo dormancy of homozygous flies for one of the two variants (*s-tim* and *ls-tim*) (Tauber et al., 2007) has been linked to the different affinity of S-TIM and L-TIM for CRY, the blue-light photoreceptor, which mediates TIM light-dependent degradation and, in turn, clock resetting (Sandrelli et al., 2007; Damulewicz and Mazzotta, 2020). In this context, the reduced photosensitivity of *ls-tim* flies has been interpreted as a light-buffering system, which protects the clock from the considerable increase in summer day length at Northern latitudes (Pittendrigh and Takamura, 1989; Pittendrigh et al., 1991; Sandrelli et al., 2007). Along the same lines, *ls-tim* homozygous flies have recently been shown to be more rhythmic than *s-tim* homozygous flies under constant light conditions, and still able to synchronize locomotor activity with temperature cycles in LL. These data support the contention that *ls-tim* has an adaptive value in more seasonal environments, potentially explaining its Northern spread by directional selection (Tauber et al., 2007; Zonato et al., 2018; Deppisch et al., 2022; Lamaze et al., 2022).

Several studies, including the present one, also point to *s-tim* - *ls-tim* effects that are independent of photoperiodic mechanisms. Firstly, the clear photoperiodic effect on dormancy induction reported in Tauber et al., 2007 for both *s-tim* and *ls-tim* Houten lines was not reproduced in our experimental setting, where mild to no photoperiodism was observed. While the reason for this discrepancy remains unclear, the experimental paradigm used to assess dormancy in the two studies was similar but not identical. More specifically, in the present study a slightly lower temperature, a narrower post-eclosion collection window of adult flies and a shorter timespan at cool temperature were utilised. Moreover, in this study, for example, *ls-tim* homozygous flies showed lower early-life fecundity and delayed egg-to-adult development at 25°C and under LD 12:12. Further, transgenic lines which differ primarily for the *s-tim* or *ls-tim* allele have been shown to exhibit circadian periods of locomotor activity of 24.2 h and 26.2 h, respectively, in LL (Peschel et al., 2006). Similarly, the overexpression of *l-tim* or *s-tim* variants leads to longer and shorter periods of locomotor activity, respectively, under DD at both 18°C and 25°C (Anduaga et al., 2019). Taken together, these data suggest that the different reproductive and developmental profiles of *ls-tim* flies may result from dampened clock oscillations – a default state even in constant conditions - and are amplified by secondary light/photoperiod-dependent mechanisms. Similarly, *per* mutants with shorter or longer circadian free-running periods (*per* ^*S*^ and *per* ^*L*^) exhibited faster and slower egg-to-adult development, respectively (Kyriacou et al., 1990; Srivastava et al., 2018). However, *per* ^*S*^ and *per* ^*L*^ mutant flies showed comparable photoperiodic response curves for dormancy induction (Saunders et al., 1989), and negligible photoperiodic effects in cold-induced coma recovery time (Pegoraro et al., 2014).

The Northward spread of the *ls-tim* allele in Europe has been suggested to be driven by directional selection, as buffering light-sensitivity of the endogenous oscillator would protect the clock itself from the dramatic changes in day length that characterise summer at high latitudes (Sandrelli et al., 2007; Tauber et al., 2007; Deppisch et al., 2022; Lamaze et al., 2022). While the adaptive value of such effects on the robustness of behavioural rhythms is clear (Deppisch et al., 2022; Lamaze et al., 2022), understanding how these dynamics translate into higher levels of reproductive dormancy under both short and long photoperiods is more difficult (Tauber et al., 2007). Long photoperiods have been shown to inhibit dormancy (Saunders and Gilbert, 1990). However, in *D. melanogaster* reproductive dormancy is thought to be induced primarily by a reduction in temperature (Emerson et al., 2009). This notion is supported by studies carried out in laboratory conditions, which have documented negligible photoperiodic responses in dormancy induction under simple rectangular light-dark cycles (Schiesari et al., 2016; Andreatta et al., 2018; Nagy et al., 2019). Yet, flies exposed to simulated late autumn and summer natural lighting conditions, which better approximate the setting in the wild, showed higher and lower proportions of dormancy compared to individuals subjected to corresponding rectangular profiles, respectively. This suggests that a photoperiodic component of dormancy induction exists also in *Drosophila* (Nagy et al., 2018).

However, it should be highlighted that core clock components – including TIM – are not solely expressed in tissues which are directly light- or temperature-sensitive. For instance, TIM and PERIOD (PER) have been found to be constitutively expressed in the follicle cells of *Drosophila* ovaries, as part of non-circadian processes (Beaver et al., 2003). Interestingly, mutants for these genes have been associated with a significant decline in fertility (number of offspring) and reduced/slowed oocyte maturation, a phenotype which recapitulates some aspects of dormancy (Beaver et al., 2003). Recently, *Drosophila* dormancy has been defined as a more general stress response to cold temperatures, as oogenesis arrest at previtellogenic stages is a common hallmark of responses to other stressors (Lirakis et al., 2018). Moreover, impairment in circadian clock functioning has been shown to significantly impinge on reproductive success in both flies and mammals (Beaver et al., 2002; Ratajczak et al., 2009; Garmier-Billard et al., 2019; Horn et al., 2019). In addition, *tim* and *per* knockdown in the prothoracic gland (PG) - which is implicated in pre-adult developmental progression - resulted in reduced steroidogenesis and developmental failure (Di Cara and King-Jones, 2016). Finally, flies overexpressing *tim* in the skeletal muscle showed extended lifespan (Hunt et al., 2019), a trait which is often associated with reduced early-life fecundity and high dormancy incidence (Schmidt et al., 2005b; Schmidt and Paaby, 2008). Thus, while the effects of TIM variants on reproductive dormancy have been linked to the different stability of TIM-CRY interactions (Sandrelli et al., 2007), any impact of S-TIM or L-TIM expression in peripheral tissues cannot be excluded and deserves further investigation.

Clock neurons and core clock components control fundamental hormonal mechanisms involved in growth, development and reproduction, such as steroidogenesis and insulin signalling (Di Cara and King-Jones, 2016; Nagy et al., 2019). Thus, it is plausible that S-TIM and L-TIM isoforms may differentially affect such processes, in a tissue-specific manner. This would, in turn, affect (or sensitize to certain environmental conditions) developmental and reproductive aspects controlled by these endocrine pathways. Interestingly, *Drosophila* natural populations with higher dormancy propensity show reduced early-life fecundity and longer egg-to-adult development (Schmidt et al., 2005b). This is in line with our findings of reduced fecundity and extended developmental time in high-dormancy *ls-tim* homozygous flies, and suggests that the genetic background at the *tim* locus might alter the flies’ developmental and reproductive trajectories. Whether the first aspect is the result of impaired/delayed gonadal development or defects in mating behaviour remains to be tested. However, the association between reduced fecundity, severe arrest/delay of ovarian growth, and prolonged developmental time observed in this study and in Schmidt et al. (2005b), possibly support the first hypothesis. We found the developmental delay characterizing *ls-tim* flies to increase from ∼13 h at 25°C to ∼36 h at 15°C. Although the temporal extension of *ls-tim* flies development can be considered temperature-dependent, it is interesting to note that it is proportional to the developmental time at the two different temperatures, resulting in a comparable % increase at 25°C and 15°C. This might suggest that the allele-specific effects on this phenotype are not temperature-dependent *per se*. On the other hand, it is worth highlighting that these data refer to pupariation time, and not eclosion. Thus, it is still possible that both *s-tim* and *ls-tim* homozygous flies maintain a circadian rhythm in eclosion, differentially regulating the timing of the final steps of metamorphosis and/or circadian gating of eclosion (Varma et al., 2019; Mark et al., 2021). Further, we hypothesize that the identified delay might be instrumental to prolong larval feeding and thus the growth phase, resulting in larger flies, a trait which is strongly selected at higher latitudes (Chown and Gaston, 2010; Stillwell, 2010), and is a feature of diapause-destined individuals in several insect species (Hahn and Denlinger, 2011). In this context, the reported ∼20% increase in weight in both male and female *ls-tim* homozygous adult flies at 15°C is likely to be the result of the ∼36 h extension in larval development. These findings are consistent with the observation that flies in which the PG (and thus the release of the hormone ecdysone) has been genetically manipulated are characterized by a ∼30 h developmental delay and a parallel increase in body weight of ∼25% (Layalle et al., 2008). It remains to be determined whether the faster development of *s-tim* homozygous flies contributes, despite the lower dormancy propensity, to the counterintuitive cline of *tim* alleles in Italy and Central Europe, with the *s-tim* allele being more abundant at Northern latitudes (Pegoraro et al., 2017). However, this seems unlikely given the inverted cline described in both Eastern United States and Spain (Pegoraro et al., 2017; Zonato et al., 2018). Given its association with the distance from the hypothesized site of origin rather than latitude (Zonato et al., 2018) *ls-tim* frequency is likely to be the product of directional and not balancing selection, at least in Europe.

In conclusion, our data show that *s-tim - ls-tim* natural alleles influence sets of intertwined life-history traits, possibly contributing to potentiate the effects of specific seasonal adaptation in temperate zones.

## Data Availability

The raw data supporting the conclusions of this article will be made available by the authors, without undue reservation.
